# Retraction: The Plausible Relationship Between Periodontitis and Glaucoma

**DOI:** 10.7759/cureus.r68

**Published:** 2023-01-26

**Authors:** Kamakshi Gupta, Navneet Kaur, Gurpreet Kaur

**Affiliations:** 1 Department of Periodontics, National Dental College and Hospital, Derabassi, Mohali, IND; 2 Department of Periodontics and Implantology, National Dental College and Hospital, Derabassi, Mohali, IND

This article has been retracted due to plagiarism of the following article: Pachiappan Arjunan (2021) Eye on the Enigmatic Link: Dysbiotic Oral Pathogens in Ocular Diseases; The Flip Side, International Reviews of Immunology, 40:6, 409-432, DOI: 10.1080/08830185.2020.1845330

The journal was contacted by Dr. Pachiappan with concerns regarding unauthorized reuse of two figures as well as multiple sections of text that have been reproduced verbatim by the authors without citation and quotation, including the article abstract, figure legends and article conclusions. Unfortunately, this duplication was not flagged by iThenticate, the plagiarism checking tool used by Cureus, and thus was not detected prior to publication. The authors did not dispute these allegations when confronted and agreed that the article should be retracted.

